# Mapping QTLs for *Fusarium* Head Blight Resistance in an Interspecific Wheat Population

**DOI:** 10.3389/fpls.2016.01381

**Published:** 2016-09-29

**Authors:** Angelica Giancaspro, Stefania L. Giove, Daniela Zito, A. Blanco, Agata Gadaleta

**Affiliations:** ^1^Department of Agricultural and Environmental Sciences, Research Unit of “Genetics and Plant Biotechnology”, University of Bari Aldo MoroBari, Italy; ^2^Department of Soil, Plant and Food Sciences, Section of Genetics and Plant Breeding, University of Bari Aldo MoroBari, Italy

**Keywords:** durum wheat, FHB resistance, genetic map, QTL analysis, SNP markers

## Abstract

*Fusarium* head blight (scab) is one of the most widespread and damaging diseases of wheat, causing grain yield and quality losses and production of harmful mycotoxins. Development of resistant varieties is hampered by lack of effective resistance sources in the tetraploid wheat primary gene pool. Here we dissected the genetic basis of resistance in a new durum wheat (*Triticum turgidum* ssp. *durum*) Recombinant inbred lines (RILs) population obtained by crossing an hexaploid resistant line and a durum susceptible cultivar. A total of 135 RILs were used for constituting a genetic linkage map and mapping loci for head blight incidence, severity, and disease-related plant morphological traits (plant height, spike compactness, and awn length). The new genetic map accounted for 4,366 single nucleotide polymorphism markers assembled in 52 linkage groups covering a total length of 4,227.37 cM. Major quantitative trait loci (QTL) for scab incidence and severity were mapped on chromosomes 2AS, 3AL, and 2AS, 2BS, 4BL, respectively. Plant height loci were identified on 3A, 3B, and 4B, while major QTL for ear compactness were found on 4A, 5A, 5B, 6A, and 7A. In this work, resistance to *Fusarium* was transferred from hexaploid to durum wheat, and correlations between the disease and morphological traits were assessed.

## Introduction

*Fusarium* head blight (FHB) is one of the most widespread and damaging diseases of wheat worldwide. Two kinds of resistance to FHB were first described by Schroeder and Christensen (1963), and they are type I (resistance to initial infection) and type II (resistance to fungal spread within the spike). A third kind of resistance has been reported as related to the accumulation of mycotoxins within the grains (Mesterhazy et al., 1999). The incidence and severity of FHB are independent from genotypes, and vary from year to year depending on climate conditions and distribution areas of the varieties, resulting in severe yield losses as well as significant grain quality damage due to the presence of mycotoxins. Decreases in production are due both to the fact that affected ears have a very shriveled grain and to the inoperability of the kernels because the responsible fungi are capable of forming numerous mycotoxins, some of which have significant impact on human and animal health, such as deoxynivalenol (DON) and zearalenone (ZEA). The presence of mycotoxins, and more specifically of *Fusarium* toxins, is the major problem in durum wheat as it is used prevalently for human consumption. FHB has been associated to over 17 different species of the *Fusarium* genus (Stenglein, 2009), but *F. graminearum* Shwabe (teleomorph *Gibberella zeae*), *F. culmorum* (W.G. Smith), and *F. avenaceum* (teleomorph *Gibberella avenaceae*) predominate depending on climate conditions. Studying the population of this fungus is extremely important because the same species can produce different mycotoxins, and the same mycotoxin may be produced from different species. Currently, there are still no methods to remove mycotoxins from food, so the only solution to reduce the risks connected to mycotoxins accumulation is to avoid the development of fungi which produce them, during both cultivation and storage phases. The development of tolerant or resistant cultivars is the only effective strategy for the control of FHB disease. Despite this, the results available to date are very few, and are largely related to hexaploid wheat where some resistant lines have been developed in Eastern countries which have been suffering from FHB epidemics for several decades (Prat et al., 2014).

In recent years, the lack of precise knowledge on the inheritance mechanism of FHB resistance has placed a major stumbling start for screening programs of wild relative collections, and for the genetic improvement of this trait in durum wheat. Resistance to *Fusarium* is a quantitative trait and, as such, is assumed to be controlled by the combined effects of several quantitative trait loci (QTL), epistasis (interaction between QTL), environment, and interaction between QTL and the environment (Zhou et al., 2002; Buerstmayr et al., 2009, 2012). Linkage analysis and association mapping are the two most commonly used approaches for QTL mapping; moreover, the availability of linkage maps based on abundant DNA markers has vastly improved the ability to identify and characterize such QTL.

Recently, the availability of sequence data in wheat allowed the detection and a wide use of single nucleotide polymorphisms (SNPs) markers for the development of genetic linkage maps (Akhunov et al., 2009). A high-density wheat SNP iSelect array comprising approximately 90,000 gene-associated SNPs that provides dense coverage of the wheat genome was developed by Wang et al. (2014) and can be efficiently used for the study of quantitative traits through the development of high-resolution maps.

The review of Prat et al. (2014) reported that the genetic basis of FHB resistance of a few tetraploid sources in the *T. durum* background has been dissected by QTL mapping and that so far, 13 QTL with small to moderate effects have repeatedly been detected on 11 chromosomes with alleles improving FHB resistance deriving from relatives and durum wheat itself (Snijders, 1990; Bai and Shaner, 1994; Mesterhazy, 1995; Mesterhazy et al., 1999; Anderson et al., 2001; Ruckenbauer et al., 2001; Buerstmayr et al., 2002; Liu and Anderson, 2003). The 3B chromosome seems to have an important role in the resistance in fact a major QTL, Qfhs.ndsu-3BS was well analyzed for FHB type II resistance (Waldron et al., 1999; Anderson et al., 2001; Buerstmayr et al., 2002; Zhou et al., 2002; Somers et al., 2003; Yang et al., 2003) while a QTL for DON resistance was fine mapped on the distal segment of chromosome 3BS linking with *Xgwm533* and *Xgwm493* as *Fhb1* (Cuthbert et al., 2006).

Several sources of resistance have been identified in hexaploid wheat. The Chinese cultivar Sumai 3 and its derivatives (Bai et al., 2003), many Japanese accessions (Mesterhazy, 1997; Ban, 2000; Rudd et al., 2001), the Brazilian Frontana cultivars (Singh et al., 1995; Mesterhazy, 1997), and germplasm from Eastern Europe as “Prag 8” (Mentewab et al., 2000) are carriers of the hallmarks of resistance to diffusion in the ear. Currently, however, durum wheat cultivars with a high resistance to FHB are still not available, although some cultivars can be exploited due to a partial resistance, which allows to partially limit the loss of production and accumulation of mycotoxins. Durum wheat also appear more susceptible to FHB compared to common wheat, due to some morphological traits able to aggravate disease development (compact spike, tendency to retain anthers inside the floret, early flowering coinciding with rainfall, and wet climate conditions; Lops et al., 1998), and its management is complicated by technical factors. Moreover, susceptibility to FHB is increasing among durum varieties due to expansion of the original cultivated area (from warm and dry Mediterranean nations to rainy regions of Western Europe) following the increasing demand for durum-derived products. The reason for the scarcity of FHB-resistant sources in durum wheat has not yet been clearly elucidated (Prat et al., 2014). A possible explanation could be that current durum wheat, mostly descending from germplasm cultivated in the warm and summer-dry Mediterranean basin, has not been exposed to relevant disease pressure (Ban et al., 2005). Moreover, attempts to transfer resistant QTL identified in *Triticum aestivum* into *T. durum* have met with limited success so far (Gilbert and Tekauz, 2000; Oliver et al., 2007; Buerstmayr et al., 2012), prevalently due to differences in ploidy levels. The use of hexaploid wheat as a resistance donor is not straight forward (Prat et al., 2014).

In the present research, we want to study the genetic basis of FHB resistance transferred from a hexaploid wheat resistant line into a susceptible tetraploid wheat cultivar. Therefore, the objectives of this work were: constitution of a recombinant inbred lines population from crossing a resistant hexaploid wheat line and the susceptible tetraploid cv. Saragolla; development of a new genetic linkage map and detection of QTL for type I and type II FHB resistance; identification of molecular markers tightly linked to resistant loci to be used in future marker-assisted selection programs or positional cloning.

## Materials and Methods

### Plant Material Constitution

A FHB-resistant bread wheat accession (02-5B-318, a breeding line derived from the resistant Chinese cv. Sumai-3 kindly provided by S.I.S., Bologna, Italy) and the FHB-susceptible durum wheat cv. Saragolla were crossed, and a total of 421 RILs were obtained by advancing random individual F_2_ plants to F_7_ generation by single seed descent. DNA extraction was performed from fresh young leaves of five pooled plants for each RIL and the parental lines as described by Gadaleta et al. (2014). Each progeny was evaluated for the presence or the lack of D genome chromosomes by using a set of 14 single band, D genome-specific gSSR markers, one mapping on the short and one on the long arm of each D genome chromosome (CFD61-1DS, GDM11-1DL,GWM261-2DS, BARC228-2DL, GWM341-3DS,GWM3-3DL, WMC720-4DS,GWM624-4DL, CFD189-5DS, GWM292-5DL, WMC749-6DS, BARC175-6DL, GWM295-7DS, and WMC94-7DL). The markers were chosen following the consensus map from Marone et al. (2012). In addition, a set of aneuploid lines derived from the hexaploid cv. Chinese Spring, including nulli-tetrasomic (NT) and di-telosomic (DT) (see references in Gadaleta et al., 2014) lines for the D genome, was used to check the specificity of each microsatellites marker. The D genome-specific SSRs were analyzed in each line, and the amplified fragments visualized by capillary electrophoresis at the 3500 Genetic Analyzer (Applied Biosystems). Thus, two RIL populations were developed, one of hexaploid and one of durum wheat.

### Field Experiments and FHB Resistance Evaluation

A set of 135 durum wheat RI Lines and the two parents (02-5B-318 and Saragolla) were evaluated for two components of FHB resistance (incidence and severity) in replicated field trials carried out in two locations and 2 years: Bologna 2012 (BO12), Bologna 2013 (BO13), and (Bari) 2013 (BA13). Field experiments were arranged in a randomized complete block design with three replicates and plots consisting of 1 m rows, 30 cm apart, with 80 germinating seeds per plot. During the growing season, 10 g of nitrogen per m^2^ and standard cultivation practices were adopted.

In Bologna, the RIL population was evaluated in field trials under natural infection occurrence, whereas in Bari, lines were tested under controlled conditions in a greenhouse located in Bari (BA) following artificial inoculation with the *F. graminearum* single-spore isolate “PH-1” (kindly provided by prof. Quirico Mighelli, University of Study of Sassari, Italy). *Fusarium* strain was grown as described by Lionetti et al. (2015). Plants were artificially inoculated by applying a uniform inoculum pressure during anthesis in the late afternoon. Twenty plants for each genotype were inoculated in each replicate choosing five spikes each, for a total of 100 spikes. Plants were sprayed with 100 mL of a distilled-water suspension of *F. graminearum* conidia (about 1.0 × 10^6^ conidia per mL); after inoculation, spikes were covered overnight with polyethylene bags to maintain high humidity. Each plot was inoculated twice, the first time when 50% of the heads within a plot was flowering, and the second time 5 days later.

*Fusarium* head blight incidence and severity were recorded at 14 and 21 days after first inoculation (in greenhouse experiments) or after flowering (in field trials with natural inoculum) on both infected and mock-inoculated (controls) wheat plants: FHB incidence was averaged as the number of infected spikes per plant, while FHB severity was averaged as the visually estimated percentage of infected spikelets per plant.

The two parents and the RI progenies were also evaluated for some morphological traits reported to be associated to resistance, including plant height, spike compactness, and awn length. Plant height and spike compactness were assessed in all the three experiments (BO12, BO13, and BA13), while awn length was detected in BO13 only. Plant height was measured in cm. Awn length was visually scored from 0 (short) to 9 (long), and spike density was scored from 0 (loose) to 9 (very compact) as reported in Buerstmayr et al. (2012). Awn length and ear compactness were visually assessed after anthesis, as this is the most susceptible developmental stage to *Fusarium* infection.

### Molecular Markers Analyses and Map Construction

The 90K iSelect array developed by Illumina CSPro^®^ (San Diego, CA, USA) and described by Wang et al. (2014) was used to survey 81,587 SNP sequences across the two parental lines and the whole RIL mapping population. Genotyping was performed on 1 μg of genomic DNA at “TraitGenetics” GmbH (Gatersleben, Germany)^1^ following the manufacturer’s recommendations as described by Akhunov et al. (2009). The genotyping assays were carried out on the Illumina iScan reader and performed using GenomeStudio software v 2011.1 (Illumina CS Pro^®^). Each marker was tested for deviation from the expected 1:1 ratio by Chi-square analysis. Linkage analysis and map construction were performed by the JoinMap software v. 4.0 (Van Ooijen and Voorrips, 2001) and the Kosambi mapping function was used to calculate map distances (Kosambi, 1944). Linkage groups (LGs) were established using a minimum LOD score of 10.0 after preliminary analysis using LOD scores ranging from 3 to 5.

### QTL Analysis

Frequency distribution of the phenotypic data were tested for normal distribution, to estimate the complexity of genetic control of the traits. Means of phenotypic traits within replicates and within experiments were calculated and used for statistical analysis. Analysis of variance (ANOVA) and correlation tests were performed to check the significance of differences among RILs and replicates using MSTATC software (v. 6.0.1). The effects of replications and genotypes were considered in the model. Data of each experiments were analyzed by two-way ANOVA and a least significant difference (*P* < 0.05) was calculated. Broad-sense heritability was estimated from variance components with the equation *H*^2^ = σ^2^_G_/[σ^2^_G_ + (σ^2^_GE_/E) + (σ^2^_e_/rE)], with σ^2^_G_, the genetic variance; σ^2^_GE_, the genotype × environment interaction variance; σ^2^_e_, the residual variance; E, the number of environments; r, the number of replicates per line (Nyquist, 1991).

The inclusive composite interval mapping (ICIM) method (Li et al., 2007) was employed for QTL mapping using QGene 4.0 software (Joehanes and Nelson, 2008). A scanning interval of 2 cM between markers and putative QTL with a window size of 10 cM was used to detect QTLs.

The number of marker cofactors for background control was set by forward regression with a maximum of five controlling markers. Putative QTLs were defined as two or more linked markers associated with a trait at a LOD ≥ 3. For main QTL effects, positive and negative signs of the estimates indicate the contribution of Saragolla and 02-5B-318, respectively, toward higher trait value.

For all traits, QTL analyses were carried out using RILs means for individual environments, as well as for the mean across environments. Besides the classical CIM analysis, a multiple trait multiple interval mapping (MTMIM) of QTL was also performed with QGene 4.0 software combining incidence and severity over years. QTL for incidence were designed as QFhi.mgb and QFhs.mgb- for severity including: Q name of the trait. name of laboratory where has been generated-chromosome location.

The proportion of phenotypic variance explained by a single QTL was determined by the square of the partial correlation coefficient (*R*^2^). Pearson phenotypic correlation coefficients were calculated among the morphological parameter and FHB resistance in each year. For the estimation of variance components and broad-sense heritability all effects were considered random.

## Results

### Constitution of an Interspecific Population

With the aim of developing a RIL population suitable for studying the genetic basis of FHB resistance in durum wheat, a resistant bread wheat accession (02-5B-318) and a susceptible durum cultivar (Saragolla) were crossed. These two parents were well characterized from the same authors in the manuscript from Lionetti et al. (2015) and differed for several traits linked to FHB resistance. These included some important passive resistance factors represented by morphological traits (plant height, spike compactness, ear, and awn length) and cell wall composition in spikes such as lignin monolignols fraction, arabinoxylan (AX) substitutions, and pectin methyl-esterification.

By evaluating all the 421 F_6_–F_7_ progenies with 14 D genome-specific gSSR markers mapping on the short and long arm of each D genome chromosome, two RIL populations were obtained: 165 RILs (39%) were classified as hexaploid as carrying all the D chromosomes, while the durum RIL population consisted of 210 lines (50%) completely lacking D chromosomes; the remaining 46 F_6_ lines still showed some chromosome segregation, in the sense that they retained 2 (67.3 %), 3 (16.3 %), 4 (10.2 %), 5 (4.1 %), or 6 (2.0 %) entire D chromosomes or chromosome arms.

### Trait Evaluation and Correlation

Analysis of variance calculated for the experiment in Bologna 2012, revealed highly significant differences (*P* < 0.001) among genotypes and replicates (blocks). Values of the parents, means, and ranges of the RIL population in each environment, genetic variance, and broad-sense heritability estimates for FHB incidence and severity are reported in **Table 1**. The parental lines had significantly different values for both incidence and severity in each environment (Lionetti et al., 2015).

**Table 1:** Mean, range, genetic variance (σ^2^_G_), and broad-sense heritability (h^2^_B_) estimates for FHB incidence (percentage of infected spikes per plant) and severity (percentage of infected spikelets per spike) in the RIL population derived from crossing FHB-resistant 02-5B-318 and FHB-susceptible cv. Saragolla, evaluated in three environments (Bologna 2012, Bologna 2013, Bari 2013).

Trait	Environments		
	
	BO12	BO13	BA13
**FHB incidence (%)**			
02-5B-318	2	0	6
Saragolla	95	97	100
Mean RIL	48	32	55
Range	(0–100)	(0–90)	(0–100)
CV	5.3		
σ^2^_G_	542.934		
h^2^_B_	0.92		
**FHB severity (%)**			
02-5B-318	10	0	2
Saragolla	80	75	90
Mean RIL	37	20	43
Range	(0–90)	(0–90)	(0–100)
CV	3.4		
σ^2^_G_	617.271		
h^2^_B_	0.82		


Disease symptoms were moderate to high in all environments, in fact mean of the RIL population ranged from 32 to 55% for incidence, and from 20 to 43% for severity. The highest disease incidence and severity were recorded in Bari 2013, probably due to a greater pathogen pressure caused by artificial inoculation and humidity condition. The broad-sense heritability estimates (genotype mean basis) for means across environments were 0.82 for severity and 0.92 for incidence, indicating the stability of the trait and that a large proportion of the observed phenotypic variation was mainly due to genotypic effect.

**FIGURE 1:**
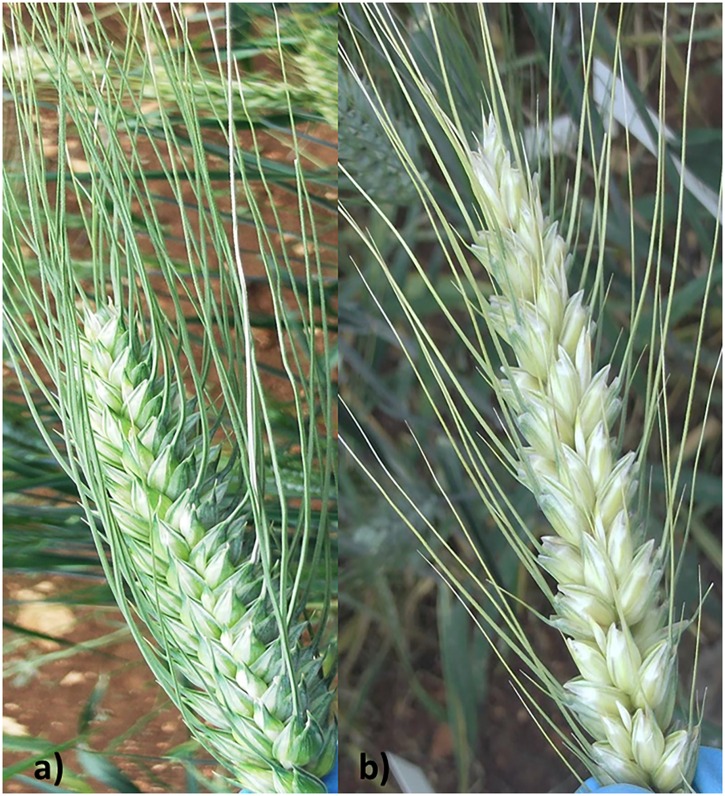
**Spike morphology of FHB-susceptible durum wheat cv. Saragolla **(a)** and FHB-resistant hexaploid wheat line 02-5B-318 **(b)****.

The two parental lines were also evaluated for morphological traits acting as passive resistance factors (plant height, spike and awn length, ear compactness); in particular, the resistant line 02-5B-318 was tall with loose spikes and long awns, whereas the susceptible cv Saragolla was short-sized, showing a dense-spike phenotype and short awns (**Figures 1a,b**). Morphological traits were used to investigate the correlations with FHB resistance.

**FIGURE 2:**
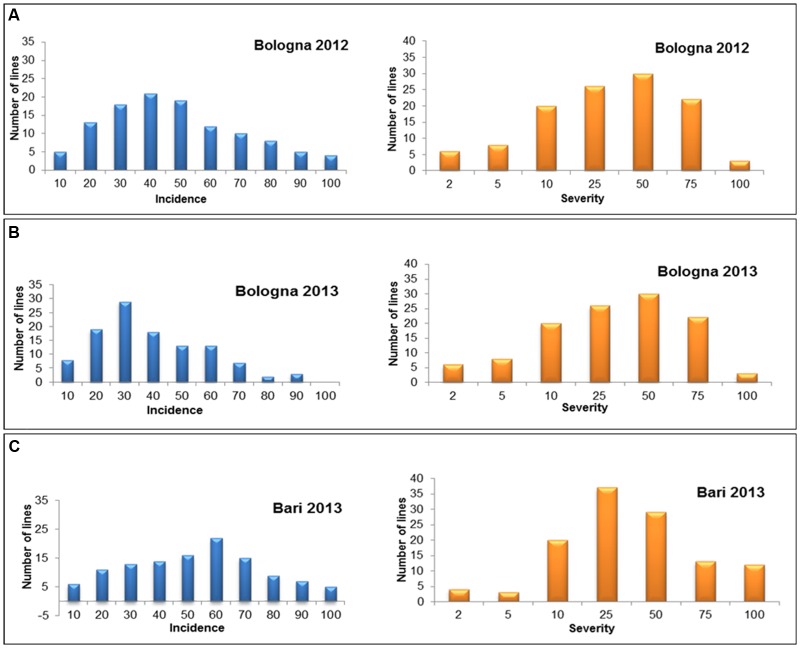
**Diagrammatic representation of frequency distribution for FHB incidence and severity in the RIL population derived from crossing the resistant 02-5B-318 line and the susceptible cv. Saragolla in the three environments of Bologna 2012 (BO12) **(A)**, Bologna 2013 (BO13) **(B)**, and Bari 2013 (BA13) **(C)****.

For both incidence and severity, the pattern of variation in the RI lines was typical of quantitative traits as shown in **Figure 2**, resembling a frequency–distribution curve with a normal trend. The RIL population showed a significant variation with high heritability also for the morphological traits plant height and spike compactness (**Table 2**).

**Table 2:** Mean, range, genetic variance (σ^2^_G_), and heritability (h^2^_B_) estimates for spike compactness and plant height in the RIL population derived from crossing FHB-resistant 02-5B-318 line and FHB-susceptible cv. Saragolla, evaluated in three environments (Bologna 2012, Bologna 2013, Bari 2013).

Trait		Environments	
	
	BO12	BO13	BA13
**Spike compactness^a^**			
02-5B-318	1.0	1.0	1.0
Saragolla	9.0	9.0	9.0
Mean RIL	5.0	4.5	5.5
Range	(1.0–9.0)	(1.0–9.0)	(2.0–9.0)
CV	5.8		
σ^2^_G_	3.762		
h^2^_B_	0.70		
**Plant height (cm)**			
02-5B-318	110	100	98
Saragolla	82	84	80
Mean RIL	83	81	80
Range	(50–142)	(52–142)	(48–140)
CV	8.3		
σ^2^_G_	402.875		
h^2^_B_	0.90		


*^a^Spike compactness was scored from 0 (loose) to 9 (very compact).*

Correlation coefficients calculated for individual environments are shown in **Table 3**. The population showed significant negative correlations (*P* ≤ 0.01) between FHB incidence and severity and plant height in the two environments where FHB resistance was evaluated under natural infections (BO12, BO13); in contrast, no significant correlation was observed in Bari 2013, where resistance was evaluated under controlled greenhouse conditions. This was probably due to the fact that an uniform inoculum pressure was applied on each line by using artificial spray inoculation. A highly significant positive correlation (*P* ≤ 0.001) between spike compactness and FHB was also evident in all the three environments. Taller lines with loose heads tended to be less infected respect to short-size and compact spikes. Awn length showed only a weak negative correlation (*P* ≤ 0.05) with FHB incidence and severity, suggesting that longer awns could slightly increase FHB resistance. No significant correlations were observed between FHB resistance and spike length.

**Table 3:** Coefficients of correlation between FHB incidence/severity and plant morphological traits in the RIL population derived from crossing FHB-resistant 02-5B-318 and FHB-susceptible cv. Saragolla, evaluated in in three environments (Bologna 2012, Bologna 2013, Bari 2013).

Traits	FHB incidence			FHB severity		
		
	Bologna 2012	Bologna 2013	Bari 2013	Bologna 2012	Bologna 2013	Bari 2013
Plant height	-0.11^∗∗^	-0.19^∗∗^	-0.14	-0.50^∗∗^	-0.12^∗^	-0.08
Spike compactness	0.23^∗∗∗^	0.17^∗^	-0.11	0.40^∗∗∗^	0.51^∗∗∗^	0.30^∗^
Awn length	-0.20^∗^	–	-	0.01	-	-
Spike length	-0.1	-0.20	0.10	0.01	0.02	0.03


*^∗^, ^∗∗^, ^∗∗∗^ Significance at *P* ≤ 0.05, *P* ≤ 0.01, and *P* ≤ 0.001 levels, respectively.*

### Genetic Linkage Map Construction

The two parental lines (02-5B-318 and Saragolla) and the whole RIL population derived from their cross were genotyped with the Illumina wheat 90K iSelect SNP assay (Wang et al., 2014). Out of 81,587 wheat sequences spotted on the array, 89.8% (73,247) could be analyzed as they gave unequivocal base-calls, whereas the remaining 10.2% (8,340) were excluded as the hybridization reactions failed (5,558) or gave an uncertain output in one or both the parental lines (2,782). A total of 62,495 (85.3%) among good-quality wheat sequences resulted monomorphic between the two wheat lines, whereas 10,752 (14.7%) were polymorphic; in particular, 10,049 (93.5%) showed a single-nucleotide substitution, while in the remaining 703 (6.5%), one of the two parental line had a null allele. However, among the polymorphic sequences, 3,050 had more than 10% missing data and 2,361 (30.6%) showed a strong segregation distortion (*P* > 0.001), thus were excluded from further linkage analyses. A final number of 5,341 SNPs were used for the development of the genetic map using a subset of 135 RILs from the mapping population.

The complete linkage map is reported in detail in the **Supplementary Table S1** and summarized in **Table 4**. Out of the total SNP markers, 4,366 (81.7%) were assembled in 52 LGs by using a LOD ≥ 20, of which 24 on A genome and 28 on the B genome, while 975 (18%) SNPs remained unlinked or were assembled in very small LGs. Chromosomal assignment of LGs was done by comparing the map with the consensus map from Wang et al. (2014) and Maccaferri et al. (2015), and from physical location of SNPs using a set of deletion lines (nulli-tatrasomic and di-telosomic lines) as reported in Colasuonno et al. (2014). No chromosome was assembled in a single LG, but the number of LGs ranged from 2 to 6, with the smallest number (2) for chromosomes 1A and 4A, and the higher number for 7A (6).

**Table 4:** Number and distribution of SNP markers in the durum wheat genetic linkage map obtained in the 135 RILs from the cross between the hexaploid 02-5B-318 line (FHB-resistant) and the durum cv. Saragolla (FHB-susceptible).

Chromosome	Linkage groups (*N*)	Total SNPs (*N*)	Map length (cM)	SNP density (*N*/cM)	SNP redundancy^∗^*N* (%)
1A	2	299	266.97	1.1	227.76
1B	3	454	280.80	1.6	338.74
2A	4	273	160.46	1.7	218.80
2B	4	436	321.41	1.4	324.74
3A	3	365	353.67	1.0	296.81
3B	4	151	212.46	0.7	102.68
4A	2	190	559.09	0.3	129.70
4B	4	385	242.28	1.6	292.76
5A	4	375	298.25	1.3	299.80
5B	5	341	249.13	1.4	282.83
6A	3	218	256.09	0.9	146.67
6B	3	85	70.34	1.2	58.68
7A	6	568	782.75	0.7	409.72
7B	5	226	173.67	1.3	184.81
Total A genome	24	2,288	2,677.28	0.9	1,726.75
Total B genome	28	2,078	1,550.09	1.3	1,585.76
Total AB genomes	52	4,366	4,227.37	1.0	3,311.76


*^∗^Redundancy refers to co-location of markers at the same genetic locus (identical and co-migrating SNP loci at 0.1 cM).*

A total of 2,288 (52.4%) SNP markers were localized on the A genome covering a total map length of 2,677.3 cM, while 2,078 (47.6%) mapped on the B genome (total length 1,550.1 cM). The two genome had approximately the same number of mapped markers. The whole map covered a distance of 4,227.4 cM, with an average chromosome length of 301.9 cM. The length of individual chromosomes varied from 70.3 (chromosome 6B) to 782.7 cM (7A), the differences in map length of this two chromosomes is due to the different number of polymorphic markers found, whereas the number of loci per chromosome ranged from 85 (6B) to 568 (7A). SNP markers were generally well distributed throughout the genomes, although some chromosomes exhibited a higher density. Values were comprised between 0.3 (on 4A) and 1.7 (on 2A) SNPs/cM. The overall SNP density was 0.9 markers/cM for A genome and 1.3 for B genome, with an average value of 1 SNP/cM.

Among the total SNPs on the map, 4,204 (96.3%) were nucleotide substitutions, whereas the remaining 162 (3.7%) showed a pattern with one null allele. In particular, out of the substitutions, 3,496 (83.2%) were transitions, whereas 708 (16.8%) were transversions. Among transitions, the four types A→G, G→A, C→T and T→C were almost equally represented (24.6%; 24.4, 26.0, and 25.0%, respectively); differently, out of the transversions, A→C, C→A, G→T, and T→G were more represented (25, 24.4, 20.9, and 27.7%) respect to A→T, T→A, G→C, and C→G (0.6, 0.1, 0.4, and 0.8%).

The linkage analysis revealed a very high number of markers that co-segregated and therefore mapped on the same locus; in particular, the percentage of SNPs showing redundancy (identical and co-migrating loci mapped within 0.1 cM), was of 75 and 76%, respectively, on the A and B genome. Chromosome 5B exhibited the maximum redundancy for SNP markers (83%), while the lower redundancy was observed for SNPs located on chromosome 3B (68%). Seven major SNP clusters (regions encountering ≥ 25 loci/0.1 cM) were identified on chromosomes 3A, 4B, 5A, and 7A, with the largest cluster located on chromosome 5A encountering 48 SNPs.

### Identification of QTL for FHB Resistance

**FIGURE 3:**
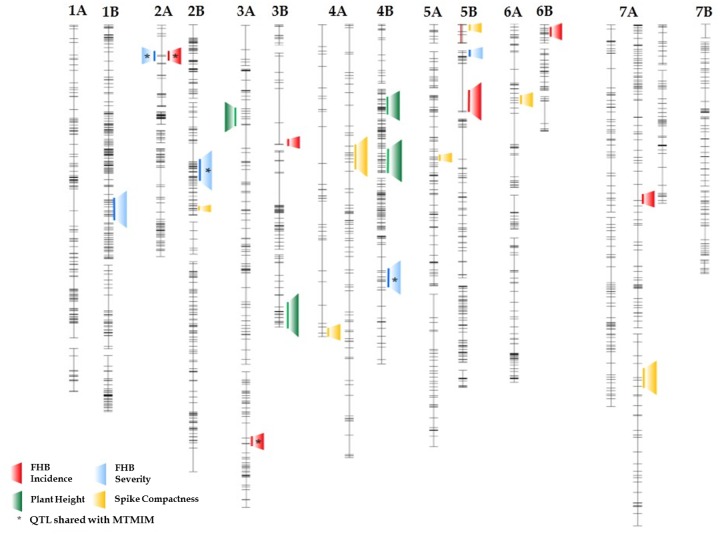
**Schematic representation of durum wheat chromosomes reporting mapped QTL (LOD ≥ 3.0) for FHB incidence (red) and severity (blue), for plant height (green) and ear compactness (yellow).** The analysis was conducted with Inclusive Interval Composite Mapping (ICIM) in the 135-RIL population derived from crossing the FHB-resistant 02-5B-318 line and the susceptible cv. Saragolla.

QTLs associated with FHB resistance were identified in the durum RIL population through the ICIM method as proposed by Zeng (1994). Putative QTLs for both FHB incidence and severity and their positions on the genetic map detected in each environment are reported in **Table 5** and **Figure 3**. QTLs with LOD values ≥ 3.0 were considered. Six genomic regions were identified for FHB incidence (type I resistance) on chromosomes 2A, 3A, 3B, 5B, 6B, and 7A accounting for a large proportion (66%) of the total phenotypic variation scored in the three environments. The amount of phenotypic variation (*R*^2^) explained by individual QTLs ranged from 8% to 12%. In particular, the major QTL (*R*^2^ = 12%) was detected on chromosome arm 2AS (LG 2A-1, closest marker IWB63138) and was significant in one single environment (BO13). Two QTL with the smallest phenotypic effect (*R*^2^ = 8%) were found on chromosomes arm 6AS (6B-3, closest marker IWA1721) and 7AL (7A-3, closest marker IWB43304). Alleles for FHB resistance were led by the 02-5B-318 line for all QTL except for that on the LG 7A-3, and LG 2B-2 where Saragolla contributed with a positive allele. The allelic effect varied in the different environments, ranging from 7.5 to 14.4.

**Table 5:** List of QTL detected for FHB incidence and severity by inclusive composite interval mapping (ICIM) in the durum wheat RIL population derived from the cross between FHB-resistant 02-5B-318 line and FHB-susceptible cv. Saragolla; for each marker, one of the co-migrating loci at the same map position is reported.

QTL name	Linkage group	Closest marker	Absolute map position (cM)	Bologna 2012			Bologna 2013			Bari 2013		
						
				Add	LOD	*R*^2^	Add	LOD	*R*^2^	Add	LOD	*R*^2^
**Incidence**												
*QFhi.mgb-2AS*^∗^	2A-1	IWB63138	28.4	9.1	3.0	11	10.4	3.0	12	–	–	–
*QFhi.mgb-3AL*^∗^	3A-3	IWB37509	49.8	–	–	–	9.7	3.5	11	9.4	3.3	9
*QFhi.mgb-3BS*	3B-2	IWB64332	68.4	–	–	–	14.0	3.1	9	–	–	–
*QFhi.mgb-5BL*	5B-2	IWB72334	45.3	–	–	–	14.4	3.1	9	–	–	–
*QFhi.mgb-6BS*	6B-3	IWA1721	20.2	–	–	–	–	–	–	8.7	3.9	8
*QFhi.mgb-7AL*	7A-3	IWB43304	109.9	-7.5	3.2	9	-8.6	3.8	8	–	–	–
**Severity**												
*QFhs.mgb-1BL*	1B-1	IWB65943	120.2	9.4	3.3	9	–	–	–	–	–	–
*QFhs.mgb-2AS*^∗^	2A-1	IWB63138	28.4	8.5	3.1	9	8.5	3.5	11	–	–	–
*QFhs.mgb-2BS*^∗^	2B-2	IWB55365	52.0	-7.8	3.9	8	-9.1	3.0	12	–	–	–
*QFhs.mgb-4BL*^∗^	4B-4	IWB48353	34.6	10.1	3.9	12	–	–	–	–	–	–
*QFhs.mgb-5BS*	5B-2	IWB816	0.0	8.0	3.0	8	6.7	3.6	7	–	–	–


*Add = additive effect denoting the contribute of the resistant or susceptible line allele. *R*^*2*^ = percentage of phenotypic variance explained by each QTL. ^∗^QTL shared with the multiple trait multiple interval mapping (MTMIM).*

QTL analysis for FHB severity (type II resistance) identified five QTL regions located on chromosomes 1B, 2A, 2B, 4B, and 5B, with a *R*^2^ comprised between 7 and 12% and an allelic additive effect ranging from 6.7 to 10.1. The QTL with the smallest phenotypic effect was mapped on chromosome arm 5BS near to marker IWB816, and accounted for 7% of phenotypic variance. Two major QTL were detected on chromosome 2BS and 4BL, respectively, close to markers IWB55365 and IWB48353, each accounting for 12% of phenotypic variation. Alleles increasing FHB resistance were led by the 02-5B-318 line at all the loci, except for QTL on 2BS for which resistant allele was led by the susceptible parent Saragolla.

Besides the classical QTL single trait analysis usually performed separately for incidence and severity, in the present work, a novel statistical method, the MTMIM was applied, in order to identify possible genetic correlations between these two traits as proposed by Da Costa et al. (2012). MTMIM analysis conducted for both FHB incidence and severity across the three environments identified nine QTL on chromosomes 1AL, 2AS, 2BS, 3AL, 4AS, 4AL, 4BL, 5BL, and 7AL (**Table 6**). Out of these, four genomic regions were shared with the classical ICIM analysis (2AS, 2BS, 3AL, and 4BL), while five QTLs were new (1AL, 4AS, 4AL, 5BL, and 7AL).

**Table 6:** List of QTL detected for FHB incidence and severity by multiple trait multiple interval mapping (MTMIM) in the durum wheat RIL population derived from the cross between FHB-resistant 02-5B-318 line and FHB-susceptible cv. Saragolla; for each marker, one of the co-migrating loci at the same map position is reported.

Chromosome arm	Linkage group	Absolute map position (cM)	Closest marker	MTMIM LOD
1AL	1A-1	161.8	IWB11516	4.1
2AS^∗^	2A-1	28.4	IWB63138	7.7
2BS^∗^	2B-2	55.2	IWB6607	5.2
3AL^∗^	3A-3	61.0	IWB8288	4.6
4AS	4A-1	63.6	IWB21309	10.7
4AL	4A-2	135.7	IWB24360	5.4
4BL^∗^	4B-4	32.8	IWB74188	5.7
5BL	5B-3	44.0	IWB36220	7.8
7AL	7A-4	106.1	IWB1277	3.8


*^∗^QTL shared with the single-trait inclusive composite interval mapping (ICIM).*

### Identification of QTL for Morphological Traits

Quantitative trait loci, estimates of QTL effects, and map position for plant height and spike compactness are shown in **Table 7** and **Figure 3**. Plant height was found to be influenced by three QTL detected on 3A, 3B, and 4B chromosomes. The QTL on 4B explained the highest quote of phenotypic variation (29%) with a LOD score of 7.1, while the QTL on 3B contributed a smaller quote corresponding to 19%. Minor loci for this trait were those found on chromosome 3A. The allele for increased plant height came from resistant parent 02-5B-318 for the QTL on 4B, whereas it was led from the susceptible parent Saragolla on chromosomes 3A and 3B.

**Table 7:** List of QTL for morphological traits (plant height and spike compactness) detected by inclusive composite interval mapping (ICIM) in the durum wheat RIL population derived from the cross between resistant 02-5B-318 line and susceptible cv. Saragolla; for each marker one of the co-migrating loci at the same map position is reported.

Chromosome arm	Linkage group	Marker interval	Map Position	Bologna 2012			Bologna 2013			Bari 2013		
						
			cM	Add	LOD	*R*^2^	Add	LOD	*R*^2^	Add	LOD	*R*^2^
**Plant Height**												
3AS	3A_2	IWB73247-IWB53051	42.0–69.3	-9.5	3.3	11	-7.4	3.3	11	-7.2	3.4	11
3BL	3B_4	IWB42624-IWB72177	19.8–54.4	-11.2	4.4	19	-9.1	2.7	13	-8.4	3.6	12
4BS	4B_2	IWB23940-IWB54727	0.0–28.7	11.8	7.1	29	11.7	6.8	29	10.2	5.5	23
4BL	4B_2	IWB65850-IWB74036	42.5–62.2	8.6	3.0	14	10.3	4.3	20	9.9	4.4	19
**Spike compactness**												
2BS	2B_2	IWB35283-IWB66885	73.4–76.0	–	–	–	1.7	2.0	9	–	–	–
4AS	4A_1	IWA3344-IWB20983	224.1–231.3	–	–	–	-1.8	2.2	10	–	–	–
4AL	4A_2	IWB617-IWA7265	267.3–295.6	–2.7	2.3	10	-	-	-	-	-	-
5AL	5A_3	IWA850-IWB72732	46.2–52.1	1.7	2.4	10	1.7	1.9	9	-	-	-
5BS	5B_1	IWB11318-IWB536	0.0–7.6	-1.8	3.4	14	–	–	–	–1.8	2.4	10
6AS	6A_1	IWA705-IWB41817	48.6–59.0	–	–	–	–	–	–	–2.8	2.4	10
7AL	7A_4	IWB26183-IWA5913	39.3–60.6	–2.8	2.7	11	–2.7	2.0	9	–2.6	2.0	9


Altogether, six different genomic regions identified on chromosomes 2B, 4A, 5A, 5B, 6A, and 7A affected spike compactness. The strongest effect was from the QTL on 5BS and 7AL, which, respectively, accounted for 14 and 11% of phenotypic variation in BO12. The increasing allele for the character was led from the susceptible durum cultivar Saragolla for all the QTL except for those on 2BS and 5AL chromosomes.

## Discussion

The spreading of sustainable agriculture in cereal crops, especially for durum and common wheat, is causing a justified farmers’ demand for appropriate genotypes to be cultivated in alternative agriculture systems. FHB is a serious concern in durum wheat, and there is an urgent need to develop resistant cultivars as managing FHB is difficult and cultural practices or chemical treatment offer only limited efficacy. While the development of resistant cultivars has been under intensive research in hexaploid bread wheat particularly during the past decade, durum wheat has received significantly less attention. An extensive collection of about 6,000 durum wheat accessions were screened for FHB resistance, none showing enhanced resistance, and a further screening survey of material from CIMMYT and ICARDA identified only five lines from a Tunisian source that exhibited moderate resistance to FHB spread (Elias et al., 2005; Huhn et al., 2012). It was accordingly speculated that durum wheat either lacks resistance genes or carries susceptibility factors and/or suppressor genes that compromise FHB resistance (Ban and Watanabe, 2001; Kishii et al., 2005; Buerstmayr et al., 2012).

Back-crossing strategies should allow transfer of desired alleles into regionally adapted elite germplasm but so far attempts to transfer FHB resistance from hexaploid wheat to durum have not been successful (Oliver et al., 2007). In the present manuscript for the first time as far we searched in literature, it has been conducted a successful experiment of transfer FHB resistance from a resistant hexaploid wheat line (02-5B-318) to a susceptible durum cultivar (Saragolla) through the constitution of a segregating RIL population. The cross of these two lines was quite difficult and led to the production of 421 F_6_–F_7_ progenies including both hexaploid and tetraploid lines, with 46 lines showing D chromosomes or chromosome arm segregation.

FHB resistance has frequently been found associated with plant morphological and developmental traits, especially plant height, spike architecture, anther extrusion, and flowering date, acting mainly as passive resistance factors (Mesterhazy, 1995). For this reason, the durum RI population was also evaluated for plant height, awn length, and spike compactness. We found a negative correlation between FHB incidence and severity and plant height, as well as a positive correlation with spike compactness in the three environments. These results agree with previous reports: particularly plant height has repeatedly been found associated with FHB severity measured in spray-inoculated experiments (Steiner et al., 2004; Draeger et al., 2007; Holzapfel et al., 2008; Buerstmayr et al., 2009, 2012; Srinivasachary et al., 2009) although no QTLs were found co-localized with those of resistance. Variation in plant height in our population was very wide, in view of differences in plant height between the tallest and shortest lines of up to 100 cm. The two parents differ also for spike compactness, which was loose in the resistant parent 02-5B-318 and very compact in the susceptible cv Saragolla. The phenotype of a characteristic durum spike differs considerably from the spike of hexaploid bread wheat. The typically compact spike phenotype of durum wheat in combination with the tendency to retain anthers inside the florets may to some extent aggravate disease development (Prat et al., 2014). The constitution of the RI lines allowed the introgression of QTL for FHB resistance in a tetraploid background, and this was demonstrated with the obtainment of lines that provide stable and enhanced resistance in field experiments. The presence of resistant alleles on the A and B genome was also reported by previous works (Buerstmayr et al., 2009), even though the role of the D-genome, which is absent in tetraploid wheat, may play a role in boosting FHB resistance (Fakhfakh et al., 2011).

A new genetic linkage map was developed in the present RIL population, and new class of markers the SNP, useful in the development and saturation of genetic maps, was applied for the detection of FHB-resistance QTLs. In the current study, we used the wheat 90K chip developed by Wang et al. (2014) to generate a high density linkage map including 4,366 SNP markers assembled in 52 LGs. This high number of LGs is due to the high LOD used for the linkage map analysis (LOD > 10), and the marker order obtained was in accordance with the consensus maps with SNP published (Wang et al., 2014; Maccaferri et al., 2015); similar results were also published by Russo et al. (2014).

The average number of mapped markers per chromosome was 311.8, the 76% of the mapped SNPs showed redundancy, i.e., identical or co-migrating loci mapped within 0.1 cM. Approximately, the same number of markers were mapped on B (2,074) and A (2,228) genome, distributed in a similar number of LGs (24 for A genome, 28 for B genome; **Table 5**). Lack of genome coverage was observed for the centromeric regions, as reported in several wheat maps (Sourdille et al., 2003; Somers et al., 2004; Torada et al., 2006).

Review by Buerstmayr et al. (2009) reported more than 100 QTL for FHB resistance in hexaploid wheat, while QTL studies in tetraploid wheat have received less attention. In the present study, we identified loci for both components of FHB resistance in durum wheat. In particular, QTL for FHB incidence, calculated with a classical ICIM analysis, were found located on chromosomes 2A, 3A, 3B, 5B, 6B, and 7A, while putative QTL for FHB severity mapped on chromosomes 1B, 2A, 2B, 4B, and 5B. QTL analysis was also conducted with the MTMIM as proposed by Da Costa et al. (2012), in order to identify possible genetic correlations between incidence and severity. The MTMIM method revealed a very useful method to discover pleiotropic effects of QTL for FHB resistance. Interestingly, nine QTLs were identified on chromosomes 1AL, 2AS, 2BS, 3AL, 4AS, 4AL, 4BL, 5BL, and 7AL: four of the regions (2AS, 2BS, 3AL, and 4BL) were in common with those found by classical ICIM analysis, while five new QTL were detected which have not been identified with the ICIM analysis (1AL, 4AS, 4AL, 5BL, and 7AL). We believe that FHB resistance of a plant is given by a combination of its response to both incidence and severity. The MTMIM method represents a convenient statistical framework to test hypotheses of pleiotropic QTL providing more details on the genetic architecture of complex traits such as FHB resistance.

All the QTL detected in our work were found reported in literature, except for the QTL located on chromosome 1A which was identified applying for the first time the MTMIM method for the study of this character. The detection of new QTL regions with MTMIM demonstrated that there are regions with pleiotropic effects on FHB resistance, and that they cannot be detected with a classical approach. Review from Prat et al. (2014) reported QTL identified on altogether 11 chromosomes, 2A, 2B, 3A, 3B, 4A, 4B, 5B, 6A, 6B, 7A, and 7B, and QTLs detected in independent studies such as QTL on chromosomes 2B, 3A, 3B, and 6B. The QTL on 3A near *XGWM-02* locus was reported in three unrelated populations using different *T. dicoccoides* accessions as resistance sources (Otto et al., 2002; Chen et al., 2007; Gladysz et al., 2007; Buerstmayr et al., 2012). Our resistance QTL located on 2A, 2B, 3A, 3B, 4A, 4B, 6B, and 7A chromosomes overlap with QTL found in hexaploid resistant sources (Buerstmayr et al., 2009), although only a chromosome arm comparison is possible as this is the first QTL map obtained with SNP markers. A certain attention should be payed to the QTL located on chromosome arm 2AS and 2BS as they co-localized with two *WheatPME1* genes physically mapped on the short arms of chromosome group 2, respectively, in 2BS1-0.53-0.75 and C-2AS5-0.78 bins (Lionetti et al., 2015). *WheatPME1* gene encodes a pectin methylesterase enzyme which modulates the degree and patterns of cell wall methyl-esterification making pectin less susceptible to degradation by pectin degrading enzymes (CWDE) produced by fungal pathogens (Limberg et al., 2000; Willats et al., 2001; Bonnin et al., 2002; Lionetti et al., 2010; Volpi et al., 2013). Pectin content and methyl-esterification in grasses has largely been associated with plant resistance to pathogens (Wietholter et al., 2003; Volpi et al., 2011, 2013; Lionetti et al., 2012; Bellincampi et al., 2014), so *pme* could be considered a good candidate for *Fusarium* resistance. In the work from Lionetti et al. (2015), expression analysis of *WheatPME1* gene conducted on the two parents of the RIL population, the resistant hexaploid 02-5B-318 line and the susceptible durum cv. Saragolla, demonstrated that in the 02-5B-318 line the level of *WheatPME1* expression tends to decrease during infection showing a onefold lower expression at 72 hpi, whereas the *pme* level doubled at 48 hpi in Saragolla. It is possible that during *Fusarium* infection, resistant plants down-regulate *WheatPME1* expression level in order to ensure a higher degree of CW methylation which would protect CW against *Fusarium* pectic enzymes. On the contrary, the susceptible line seems to induce an increased level of methyl-esterification in order to help the fungus to disassembly CW and penetrate host cells. All these results candidate *pme* gene as a susceptibility factor in durum wheat against *Fusarium* infection. Our data are supported also by Garvin et al. (2009) who mapped a QTL that increased FHB susceptibility on chromosome 2A of the *T. dicoccoides* line “Israel A.”

## Conclusion

The present work important results, have been assessed: we reached a better knowledge of the genetic basis of FHB resistance, new resistant QTLs were detected and resistance genes were transferred from hexaploid to durum wheat, with the development of durum lines useful in new advanced breeding programs.

## Author Contributions

AnG, AB, designed the experiment and developed the mapping population. AgG, DZ, SG, performed the experiments. AnG, AB supervised the overall study and along with AgG wrote the manuscript. All the authors read and approved the final manuscript.

## Conflict of Interest Statement

The authors declare that the research was conducted in the absence of any commercial or financial relationships that could be construed as a potential conflict of interest.

## Footnotes

1 http://www.traitgenetics.de
